# Harnessing Daily Routines for Early Detection of Cool Executive Delays: Validating the EFORTS Questionnaire

**DOI:** 10.3390/children11111281

**Published:** 2024-10-24

**Authors:** Carmit Frisch, Sara Rosenblum

**Affiliations:** The Laboratory of Complex Human Activity and Participation (CHAP), Department of Occupational Therapy, Faculty of Social Welfare & Health Sciences, University of Haifa, Haifa 3498838, Israel; carmitfr@shaanan.org

**Keywords:** daily routines, executive control, early detection, ecological validity, parental questionnaires

## Abstract

**Bakground**: The early identification of executive function (EF) delays should involve parental reports on children’s executive control in daily activities, capturing both “hot” and “cool” executive functions. This study aimed to revalidate the reliability and validity of the Executive Functions and Occupational Routines Scale (EFORTS) and examine whether it represents both hot and cool EFs **Methods**: Parents of 971 children (469 boys) aged 3 to 11 years (M = 6.08, SD = 1.8) completed the EFORTS and the Behavior Rating Inventory of Executive Functions/Preschool version (BRIEF/P). Exploratory and confirmatory factor analysis led to re–reestablishing the EFORTS daily functional routines with high internal consistency values (0.82–0.93). **Results**: Significant correlations were revealed between the EFORTS daily function subscales and various BRIEF/P “hot” and “cool” executive scales. Again, divided into three executive subscales, the EFORTS items showed medium-high internal reliability (α = 0.79–0.93). Significant age-related differences were demonstrated in the children’s EFs in three age groups: 3.0–5.11, 6.0–7.11, 8.0–11.11. **Conclusions**: The results suggest that EFORTS effectively assesses children’s executive control over daily functions, capturing both “cool” and “hot” EFs, making it a reliable tool for early detection of executive delays prior to formal schooling.

## 1. Introduction

Executive functions (EFs) are mental abilities that enable children and adults to gain self-control over their daily functioning, behavior, movement, and speech [1]. Using EFs is an effortful process needed mostly in situations with novel or unanticipated challenges, when it would be insufficient or impossible to go on “autopilot” or rely on instincts [2]. Several neurodevelopmental disorders involve inherent delays in EFs [3], attributed to causing many daily functional struggles [4]. Executive dysfunction in early childhood may lead to math and reading difficulties throughout school [5], more restricted play activities, and more frequent disruptive interactions [6]. More basically, without early detection and intervention, children with executive delays may struggle even to manage daily activities, such as following the sequence of expected activities during morning and evening routines [7]. Therefore, early identification of delayed EFs, leading to early intervention, may result in significant future benefits [8]. We are searching to determine how to identify delayed EFs as early as possible.

An optimal measure for identifying EF delays in young children should reflect real-world, everyday situations that demand various EF skills, across multiple settings [1]. Since parents observe their children’s daily behavior in these settings, parental questionnaires can provide valuable insights into children’s executive control outside of clinical or laboratory environments [9]. Additionally, early EF assessments should aim to capture all relevant EF components, as more than 33 concepts have been associated with EFs [1]. Common components include behavioral and cognitive inhibition, working memory (WM), emotional regulation, flexible planning, problem-solving, initiation, shifting, and monitoring [10].

Researchers often categorize these components into “cool” and “hot” EFs. Cool EFs include cognitive inhibition, WM, and flexibility, which support cognitive control in neutral, affective situations. In contrast, hot EFs, such as impulsivity, hyperactivity, and emotional regulation, come into play in motivationally significant situations [11,12]. Parents often prioritize addressing hot EF delays (e.g., hyperactivity and impulsivity) because they perceive these issues as more disruptive [7,13]. However, cool EF delays, like inattentiveness and WM difficulties, are equally important for enabling independent, efficient functioning in daily routines [14,15]. Cool EFs also help children respond appropriately to varied and stressful situations, potentially reducing anxiety and preventing mental health issues later in life [15].

Diamond [2] attributed all these EFs to three core “baskets” most required for executive control. The first “basket” includes cool (inattentiveness) and hot (hyperactivity and impulsivity) aspects of cognitive and behavioral inhibitory control. Then Diamond assigned two baskets for cool EFs: WM and flexibility. Inhibitory control, WM, and flexibility [2] develop gradually during childhood: Inhibition emerges first, and flexibility, a more sophisticated ability, develops later [1].

Cool and hot EFs tend to perform better with age. The most accelerated development observed at age 5 years [16]. Both cool and hot EFs continue to develop significantly in middle childhood (ages 6–12 years), after formal schooling begins and elevates academic and social demands [17]. However, the maturation process during this critical period remains unclear. While some evidence suggests that working memory, a cool executive function, stabilizes around the age of 8 years, hot executive functions continue to improve into adulthood. As a result, some researchers propose distinct developmental trajectories for cool and hot EFs [17].

Several parental questionnaires, such as the Behavioral Rating Inventory of Executive Function-Preschool version (BRIEF-P [18]), Ratings of Everyday Executive Functioning (REEF [19]), the Childhood Executive Functioning Inventory (CHEXI [20]), and Executive Functions & Occupational Routine Scale (EFORTS [14]), were designed to detect EF delays by evaluating children’s performance in everyday situations. However, most questionnaires focus on behavior and do not fully capture complex routines or tasks that require higher-order EFs. Additionally, many questionnaires are limited to very specific age ranges or fail to measure EFs across various environments or over time [19,21]. As a result, there is a need for tools that assess both cool and hot EFs across multiple domains and developmental stages [22].

Although one of the above questionnaires aims to assess children’s daily functioning in everyday contexts, it focuses on highly specific tasks that are only relevant to a narrow age range. For example, the activities it includes, such as keeping secrets, playing ‘hide and seek’ without cheating, or ‘I spy’ without revealing the chosen item, are typically performed by children aged 3–5. Thus, it limits the analysis of the children’s self-control along a continuum or multiple steps in daily routines during childhood. Possibly due to this focus, the questionnaire tends to correlate with tools that measure isolated cool EFs in computerized or play tasks that do not reflect complex EFs in real-world situations [19]. The questionnaires that focus on general behavior capture mainly hot EF delays [21] Therefore, they correlate mostly with tools that measure behavioral disruptions rather than EFs [9].

Thus, further research should develop new parental questionnaires or tailor the existing ones to focus on children’s self-control across multiple age-related functioning domains while applying the widely different hot and, especially, cool EF components [22]. Describing how children control themselves during full routines typical of their age may allow a comparison of how executive control develops throughout early childhood until the threshold of adolescence.

The EFORTS’s items were initially developed to capture both cool and hot EFs [14] across everyday routines which young children are involved with: Morning and evening routines, play and leisure routines, and social routines [7]. However, EFORTS’ factors have not yet reflected its underlying executive scales. The functional activities included in the EFORTS items may occur in various contextual environments, such as home (morning/evening routines), the educational system, and any place in the community (play/leisure and social routines). As suggested in the literature [22], parents completing the EFORTS are invited to score their children’s executive control related to the children’s personal contexts, preferred type of play, or involvement with their specific partners. Items that aim to capture cool EFs ask about constructed routines requiring one or more cognitive capabilities: Cognitive inhibition (attention), WM, and flexibility. For example, parents rate whether their child “solves problems that arise while performing an activity (e.g., when missing cutlery during the meal or when pajamas are in the wash).” Hot EF items focus on social routines, which are less structured and demand mainly the management of behavioral inhibition (impulsiveness/hyperactivity) and emotional regulation. For example, an item may ask whether the child, “when there is a conflict with a friend, thinks of multiple responses before responding (e.g., “says it bothers him/her or calls for help”; [14]).

Relying on Barkley’s model for EFs [1], the EFORTS items were extracted to capture Diamond’s three core EFs and also emotional regulation [14]. Our earlier study established that EFORTS functional routines among children aged 3 to 11 years relate to their executive control throughout childhood. The study examined how children perform in the EFORTS routines with the required increase in independence levels [7].

Considering the above described literature, our current study aimed to (1) reestablish the EFORTS functional routines subscales and internal reliability using a much larger sample than in the earlier study, (2) examine whether the EFORTS functional routine subscales would indeed correlate with the varied BRIEF-P EF subscales, (3) yield EFORTS executive subscales using Diamond’s theory (inhibition, WM, and flexibility) and establish their internal reliability, and (4) examine age-groups differences for the EFORTS functional and executive subscales and establish new cutoff scores for those subscales for ages 3 to 11 years. Based on the above literature regarding notable milestones in EFs development along early and middle childhood, we examined whether there are significant distinct differences between the following age groups: 3.0–5.11 (preschool period years), 6.0–7.11 years (first years at school), and 8.0–11.11 years (more advanced school years).

## 2. Materials and Methods

### 2.1. Participants

A sample of 971 typically developing children (469 boys, 502 girls) aged 3 to 11.11 years (M = 6.11, SD = 1.83) was recruited using snowball sampling, where the principal investigator distributed the questionnaire in the courses she taught, and the participants continued to share it within their communities all around the country. Neurological diagnoses were ruled out through the demographic questionnaire, where parents were asked to indicate whether they had ever sought developmental or medical assessments for their children, whether their children had been diagnosed with neurological conditions such as ADHD, DCD, SMD, ASD, language delay, cerebral palsy, or other diagnoses, and whether they had been diagnosed with emotional or behavioral conditions such as anxiety, depression, oppositional defiant disorder, etc.

### 2.2. Measures

#### 2.2.1. Demographics

Background variables collected included gender, origin country, native language, socioeconomic status, mother’s and father’s education, reports of past hospitalization, medical treatment, referral to child developmental centers, and functional concerns.

#### 2.2.2. Executive Functions and Occupational Routines Scale (EFORTS) [14]

EFORTS is a parent-reported tool designed to assess the manifestation of executive functions (EFs) in children’s daily routines [14]. EFORTS focuses on both “cool” (e.g., distractibility, working memory, cognitive flexibility) and “hot” (e.g., behavioral inhibition, emotional regulation,) executive functions, which are crucial for managing tasks such as morning routines, play, and social interactions. The scale consists of 30 items, divided into subscales based on everyday functional routines, scored from 1 (never) to 5 (always) with higher scores indicating better executive control. For example, parents are asked related the morning routine context whether their child 1. Initiates morning activities such as getting dressed, brushing teeth. 2. Persists at an appropriate pace without needing a reminder from an adult. See [14] and the Supplementary Materials for more details.

EFORTS has demonstrated high internal reliability (α = 0.82–0.93), and its ecological validity allows it to measure EFs in real-world contexts [14].

#### 2.2.3. Behavioral Rating Inventory of Executive Function (BRIEF) [23] and BRIEF-Preschool Version (BRIEF-P; [18])

The BRIEF [23] and BRIEF-P [18] assess behavioral manifestations of EFs. Respectively, these questionnaires include eight and five executive subscales that are summed to create a global executive composite (GEC). The questionnaires share the inhibition, shifting, emotional control, and WM subscales. The questionnaire items are scored from 1 (never) to 3 (often); higher scores reflect higher difficulty levels. Using age and gender norms, the subscale and GEC scores are transformed to t-scores; t-scores above 65 (SD = 1.5) classify the child with a clinical EF delay [24].

### 2.3. Procedure

This Ethics Committee of the University of Haifa approved this study (registered 206/12). The study’s goal was published to participants through WhatsApp groups and through academic courses nationwide. Participants who expressed interest in the study signed informed consent, filled out the research questionnaires, and returned them to the researcher. Due to budget constraints, only 181 parents completed the BRIEF/P questionnaires.

### 2.4. Statistical Analysis

Statistical analyses were conducted using IBM SPSS 25 and AMOS 27 software. Descriptive statistics characterized the study population. Exploratory Factor Analysis (EFA) was conducted using AMOS, and Confirmatory Factor Analysis (CFA) was performed with SPSS. Internal reliability of the factors was tested using Cronbach’s alpha (α). In the analysis conducted using AMOS software, the division of the EFORTS’ items based on routines was strong and surpassed other divisions. Therefore, the further division of these items into executive subscale was theory-driven, and we assessed the internal reliability of the factors.

Correlations between the EFORTS and the BRIEF-P were tested using Spearman’s rank correlation coefficient. We divided the EFORTS’s items using Diamond’s theory [2] and examined the new factors’ reliability using Cronbach’s α to reveal the EFORTS executive subscales.

Age-group differences in the children’s EF and everyday routines subscale scores were tested using multivariate analysis of variance (MANOVA), followed by the Bonferroni post hoc test. We used one-way analysis of variance (ANOVA) to examine differences in the EFORTS final score. The functional routines’ averages and standard deviations were computed, and new cutoff scores were calculated with 1.5 standard deviations below the mean score of each routine.

## 3. Results

As shown in Table 1, most participants were at middle socioeconomic levels or above; the mothers‘ years of education ranged between 12 and 25 years (M = 16.61, SD = 2.15); and the fathers’ years of education ranged from 8 to 28 years (M = 16.14, SD = 2.61). No significant between-group difference was found for the mothers’ or fathers’ education years, gender, and socioeconomic status.

### 3.1. Reestablishing EFORTS Daily Routines Scales: Construct (Structure) Validity

**Figure 1:**
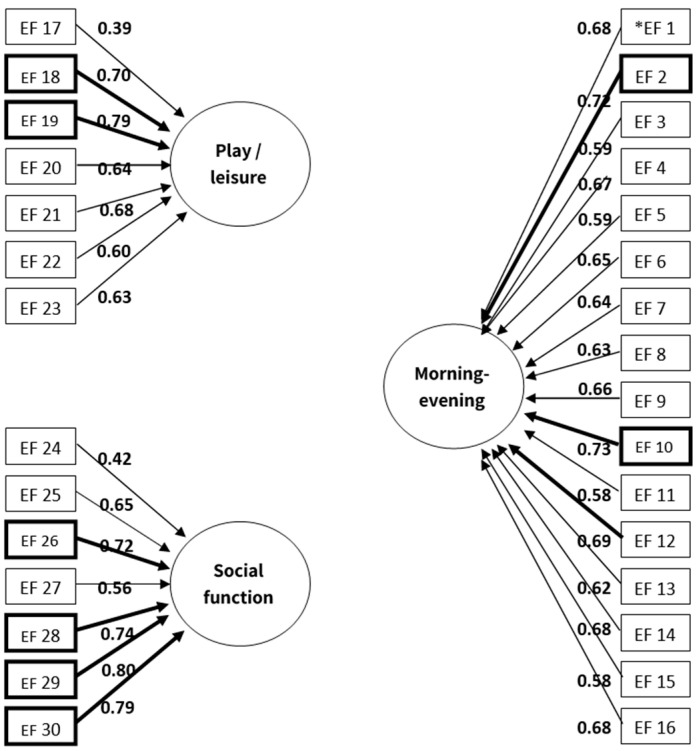
The EFORTS item distribution according to daily routines: Factor analysis and loading indices. * EF = EFORTS item. *p* < 0.001.

A CFA was conducted to reestablish the EFORTS questionnaire construct validity, dividing the EFORTS items into three factors according to previous results [14]. The analysis, using the AMOS software, presented good fit indices divided by three factors according to the questionnaire’s three distinct routines with eigenvalues > 1 comprised of the 30 items (Figure 1). The three factors yielded a cumulative percentage of variance of 51.08% with an internal consistency of α = 95. The three factors, as well as the internal consistency reliability, measured by the coefficient alpha of each factor, were as follows:

1. The first factor, morning and evening, included 16 items and accounted for 36.6% of the variance with α = 0.85. 2. The second factor, play and leisure included 7 items and accounted for 8.8% of the variance with α = 85. 3. The third factor, social participation included 7 items and accounted for 5.66% of the variance with α = 0.93.

In Figure 1, the items with the highest load values are bolded. Interestingly, the bolded items in the morning/evening and play/leisure routines (Items 2, 10, 18, and 19) reflect the children’s ability to manage themselves efficiently using WM. Essentially, they hold in their minds the rules and sequences to manage themselves toward the best consequences. The bolded items in the social routine reflect the children’s ability to inhibit their response and regulate their emotions.

### 3.2. EFORTS Concurrent Validity with the BRIEF/P

Using Pearson coefficients, significant moderate to strong correlations were found between the Brief/P scale scores (except for the shifting scale) and the EFORTS routines. Table 2 illustrates that lower score in the Brief/P scales (i.e., better EF) correlated with higher EFORTS scores, indicating the children’s more efficient management of daily routines. Moreover, compared to the BRIEF/P scales, the EFORTS morning/evening and play/leisure routines correlate significantly more with the WM and planning/organization scales.

### 3.3. EFORTS Executive Scales, Internal Reliability and Age-Related Differences: Construct (Structure) Validity

When the questionnaire items were divided according to Diamond’s three executive function scales [25], a high internal reliability was found for each component (α = 0.79–0.90). Table 3 illustrates the high number of items presenting WM alone in the evening/morning routines.

The executive subscale scores differed significantly between the younger and older age groups. According to the Bonferroni post hoc test for all three EFs (inhibition, WM, and mental flexibility), the children’s scores in all three executive scales improved with age. Table 4 illustrates the age-group differences in the children’s EFORTS executive scale scores.

### 3.4. EFORTS Main Factor Cutoff Scores

Averages and standard deviations were calculated for each routine, and the questionnaire’s final score to determine current cutoff scores according to three age groups: (a) 3 years, 0 months to 5 years, 11 months, (b) 6 years, 0 months to 7 years, 11 months, and (c) 8 years, 0 months to 11 years, 11 months. As shown in Table 5, cutoff scores for all daily routines increased with age.

The results of the MANOVA indicated significant differences between the age groups, F_(3,1960)_ = 13.31, *p* < 0.001, η_p_^2^ = 0.04. Post hoc Bonferroni tests showed that in morning/evening routine, the 6.0–7.11 and the 8.0–11.11 age groups scored better than the youngest group, 3.0–5.11 years. In play/leisure and social routines, the subscale scores increased as the group ages increased, indicating the older children’s better executive control of those routines. Additional ANOVAs related the same tendency for the EFORTS final score, which was higher with increased age, F_(2968)_ = 39.03, *p* < 0.001, η_p_^2^ = 0.08.

## 4. Discussion

The findings of this study highlight the reliability and validity of the EFORTS parental questionnaire in assessing both hot and cool EF delays among children in daily occupational contexts. With internal reliability values ranging from 0.82 to 0.93 across the three functional routine subscales, the results confirm the tool’s robustness in a large sample of 971 children aged 3 to 11 years, addressing the study’s first objective. Previous research has demonstrated the importance of structured routines for optimal child development, emphasizing the role of predictability and consistency in promoting executive control [26]. The EFORTS, with its focus on real-world daily routines, offers a more ecological assessment tool compared to existing EF measures [27]. By using EFORTS, clinicians may be able to enhance children’s executive control in everyday routines, which could serve as a protective factor, particularly for children in high-risk environments [26].

When exploring the EFORTS’ concurrent validity with the BRIEF/P, our study shows that the 30 EFORTS items capture both cool and hot executive functions (EFs). Capturing cool EFs in early childhood is challenging due to their extended developmental trajectory [18]. However, the importance of cool EFs’ early identification has prompted additional researchers to attempt apprehending them alongside hot EFs [18,19]. The utility of EFORTS lies in identifying deficiencies in cool EFs during common everyday routines in childhood, thus, better guiding targeted interventions Additionally, the highest correlation between EFORTS’ social routine and the BRIEF/P inhibition subscale (reflecting behavioral inhibition/impulsiveness) demonstrates its effectiveness in assessing also hot EFs through daily functional routines [12].

The CFA performed for Goal 1 demonstrate similar tendencies of certain hot or cool EFs required in non/constructed activities in a different method: Figure 1, with EFORTS items distributed according to daily routines, shows that the four items with the highest load values in the morning/evening and play/leisure routines are those formulated to extract WM. The four items receiving the highest load values for the reestablished social routine, however, are those worded to reflect behavioral inhibition (hyperactivity, impulsiveness), and emotional regulation. These results are supported by earlier studies linking hot EFs to affective conditions [11], that involve deliberate modulating approach [11] as required in social contexts. Current outcomes also align with our previous study and strengthen the connections between cool EFs and more structured routines with inherent multistep activities [14]. The parents’ unique insights into their children’s self-control capabilities during daily structured routines may be more indicative of how children likely gain self-control in a classroom setting with competing goals and distractions [9].

The third objective of this study was to establish the EFORTS executive subscales to guide more effective clinical interventions. Using Diamond’s [2] theory, confirmatory factor analysis (CFA) revealed three executive subscales—inhibition, WM, and flexibility—all demonstrating high internal consistency (α = 0.79–0.90). These subscales are consistent with findings from several other studies, identifying inhibition, WM, and flexibility as core executive components during early and middle childhood [5,6,11]. The combination of EFORTS cutoff scores with performance-based tools for early identification of EF delays, particularly cool EF delays, which are less observable in natural environments [14,27], may enhance early intervention efforts. Early interventions could help children develop a wide range of EF skills, which are crucial for managing daily routines and setting them on a path toward long-term success [3,25].

Previous research has shown that improving cool EF functions can significantly enhance academic performance across elementary and secondary education [28]. Future studies should investigate whether interventions focusing on improving cool EFs through daily functioning lead to gains in other functional domains, such as activities of daily living and play/leisure routines.

The fourth objective of this study was to examine age-group differences in EFORTS executive and functional subscales to illustrate the improvement as children age [16]. Our results align with previous research showing significant EF improvements throughout childhood, with the most rapid development occurring between the ages of 3.5 and 6 years relative to even 1 year later (6.0–7.0 years) [28]. In our study, we observed more pronounced changes between the youngest age group (ages 3.0–5.11) and the two older groups (ages 6.0–7.11 and 8.0–11.00) in the inhibition and flexibility subscales. These findings are supported by existing literature that describes inhibition as the first EF to develop, followed by WM and flexibility [1,25]. The simultaneous use of two sub-scales’ types, reflecting two different theoretical dimensions, within the same questionnaire contributes a modest understanding to the EFs’ development across childhood. While the division based on executive function subscales revealed significant differences between all three age groups, the division of the questionnaire items according to daily routines yields a slightly different result. As a significant improvement trend was observed across the three age groups in play/leisure and the social routines, in the morning-evening routine, a significant difference was noted only between the youngest age group and the other two age groups. Given the literature linking structured routines to cool EFs as cognitive inhibition, WM and flexibility [14], it can be argued that indeed significant maturation of cool EFs occurs at the onset of middle childhood [17], while hot EFs continue to develop more linearly.

Overall, this study demonstrates the utility of parental questionnaires, such as EFORTS, in identifying both hot and cool EF delays by examining children’s executive control in daily routines. The study’s strengths include the large sample size, covering three key age groups from early childhood through adolescence, allowing for the early detection of executive delays. However, several limitations must be considered. First, the study was conducted with a national (local) sample, and parental expectations regarding routine management may vary across cultures. Therefore, further validation of the EFORTS in diverse cultural contexts is necessary. Additionally, factors such as strict schedules, socioeconomic status, and parental expectations for independence may bias parental perceptions of their child’s abilities. Thus, future studies should combine EFORTS data with clinical observations to provide a more objective assessment.

## Figures and Tables

**Table 1:** Demographic characteristics: Comparison between age groups (*N* = 971).

**Variable**		**Age Group (Years.Months)**			***F*_(4,91804)_**	***p***	**η_p_^2^**
		3.0–5.11 (*n* = 503)	6.0–7.11 (*n* = 292)	8.0–11.11 (*n* = 176)			
**M (SD)**							
Mother’s education (years)		16.62 (2.10)	16.76 (2.33)	16.76 (2.33)	1.93	0.145	0.004
Father’s education (years)		15.93 (2.98)	16.11 (2.74)	16.11 (2.74)	0.37	0.694	0.001
			*n* (%)				
Gender	Boys	231 (45.9)	152 (52.1)	86 (48.9)	2.81	0.196	0.004
	Girls	272 (54.1)	140 (47.9)	90 (51.1)			
Socio-economic status	Low	17 (3.4)	10 (3.4)	4 (2.3)	3.87	0.056	0.006
	Mid	201 (40.0)	115 (39.4)	58 (33.0)			
	High	285 (56.7)	167 (57.2)	114 (64.8)			

**Table 2:** Correlations between the BRIEF/P subscales and the EFORTS routines (*n* = 178).

	BRIEF/P Subscale				
EFORTS Routine	Inhibition	Emotional Control	Working Memory	Plan/Organization	GEC
Morning/evening	−0.27 ***	−0.24 ***	−0.49 ***	−0.48 ***	−0.42 ***
Play/leisure	−0.39 ***	−0.24 ***	−0.49 ***	−0.39 ***	−0.44 ***
Social	−0.43 ***	−0.34 ***	−0.35 ***	−0.32 ***	−0.42 ***
Total	−0.44 ***	−0.34 ***	−0.54 ***	−0.48 ***	−0.52 ***

BRIEF/P = Behavioral Rating Index of Executive Function/Preschool version; GEC = Global Executive Composite. *** *p* ≤ 0.001.

**Table 3:** EFORTS items division into EF subscales by number or items.

Routine	Subscale *n* (Item Number)		
	Inhibition	Working Memory	Flexibility
Morning/evening	4 (6, 8, 14, 16)	9 (1–4, 7, 9–12)	3 (5, 13, 15)
Play/leisure	1 (23)	3 (18–20)	3 (17, 21, 22)
Social	3 (26, 29, 30)	2 (25, 27)	2 (24, 28)
α	0.88	0.9	0.79

**Table 4:** Age-groups differences in EFORTS executive function subscales.

Subscale	Age Group (Years.Month)			Age Affect	
	3.0–5.11 (*n* = 503)	6.0–7.11 (*n* = 291)	8.0–11.11 (*n* = 176)	*F*_(2,966)_	η_p_^2^
		M (SD)			
Inhibition	3.41 (0.60)	3.61 (0.56) *** a	3.76 (0.61) *** b * c	26.21 ***	0.05
Working memory	3.82 (0.55)	4.02 (0.50) * a	4.17 (0.53) *** b *** c	31.14 ***	0.06
Flexibility	3.66 (0.62)	3.84 (0.57) *** a	4.00 (0.62) *** b * c	22.81 ***	0.04

Difference between groups: a = between 3.0–5.11 and 6.0–7.11; b = between 3.0–5.11 and 8.0–11.11; c = between 6.0–7.11 and 8.0–11.11. * *p* = 0.05; *** *p* ≤ 0.001.

**Table 5:** EFORTS cutoff scores divided according to daily routines and to final score.

Routine	Age Group (Years.Months)					
	3.0–5.11 (*n* = 503)		6.0–7.11 (*n* = 292)		8.0–11.11 (*n* = 176)	
	M (SD)	Cutoff	M (SD)	Cutoff	M (SD)	Cutoff
Morning/evening	3.44 (0.66)	2.45	3.67 (0.62)	2.74	3.80 (0.69)	2.76
Play/leisure	3.97 (0.55)	3.14	4.17 (0.50)	3.42	4.33 (0.52)	3.55
Social	3.61 (0.60)	2.71	3.78 (0.62)	2.85	3.94 (0.60)	3.04
Final	3.67 (0.50)	2.92	3.87 (0.47)	3.16	4.03 (0.50)	3.28

## Data Availability

The original contributions presented in the study are included in the article/Supplementary Materials, further inquiries can be directed to the corresponding author.

## Supplementary Materials

The following supporting information can be downloaded at: https://www.mdpi.com/article/10.3390/children11111281/s1 (EFORTS questionnaire).
