# Supplementary material for: Harnessing Daily Routines for Early Detection of Cool Executive Delays: Validating the EFORTS Questionnaire
Source: Children (Basel). 2024 Oct 24;11(11):1281. doi: 10.3390/children11111281 (PMC11592428; doi:10.3390/children11111281)
Supplement: Supplementary file 1 [file children-11-01281-s001.zip › children-3241665-supplementary.pdf]

Executive Function and Occupational Routines Scale (EFORTS)

Frisch & Rosenblum (2014)

Name of child:___________ Date: _______ Birth date:_______ age:_______

In everyday life we and our children perform varied activities throughout the day. The purpose of this questionnaire is to evaluate your child's executive control during his daily routines. The following items ask how well he/she performs activities during key daily routines, such as morning and evening routines, play and leisure, and social routines. Please read the following questions and mark √ below the most suitable answer in reference to your child's performance.

Morning and Evening routines *Who is with your child during these routines?* ________

**In the morning - evening routine context, please specify to what extent your child:**	**Never**	**Seldom**	**Some-times**	**Often**	**always**
1. Initiates morning activities such as getting dressed, brushing teeth					
2. Persists at an appropriate pace without needing a reminder from an adult					
3. Remembers the sequence of activities to be done in the morning in their correct order					
4. Gets organized according to the rules of the house, e.g. washes hands after using the toilet, and removes plate from the table when finishes eating					
5. Solves problems that arise during activity, e.g. searches independently when cannot find needed objects.					
6. Performs only activities that promote dressing and preparing to leave home, does not deal with other things that sees or hears, e.g. ignores the television					
7. Maintains the quality of execution, e.g. checks clothes are worn the right way round					
8. Tends to complete actions without stopping in the middle and moving on to others					
9. Initiates execution of evening activities, e.g. initiates putting on pajamas					
10. Continues an activity at an appropriate pace, without being reminded by an adult					
11. Remembers the sequence of evening activities in the correct order					
12. Is organized in accordance with the house rules, e.g. helps clears up the table, puts dirty clothes in the correct place					
13. Solves problems that arise while performing an activity, e.g. when missing cutlery during the meal, or when pajamas are in the wash.					
14. Performs only activities that promote preparation for sleep, refrains from dealing with other things that sees/hears e.g., ignores television					

© All rights reserved

15. Refers to the quality of performance, e.g. checks his pajamas are not inside out					
16. Tends to complete actions without stopping in the middle and moving on to others					

Play and leisure routine. *Who's with your child during this routine?* _________

In the Context Play & Leisure routine, please specify to what extent your child:	Never	Seldom	Some-times	Often	always
17. Initiates performance of activities, e.g. chooses a game					
18. Manages to play at a suitable pace (not too fast or too slow)					
19. Plays a game according to the correct steps and in the correct order					
20. Plays according to the rules of the game, e.g. waits for his turn					
21. While playing a specific game performs only tasks related to it, avoids walking around the room/touching and engaging in other games					
22. Stops to think before playing, e.g. in a construction game, imagines what the construction will look like, chooses a design, or plans before beginning					
23. Finishes one game before moving to another					

Social routine *Who's with your child during this routine?* _________

In the Context social routine, please specify to what extent your child:	Never	Seldom	Some-times	Often	always
24. Initiates social interaction, e.g. may invite a friend over					
25. Learns from negative social experiences e.g. when does something that upsets a friend, refrains from repeating it					
26. Refrains from excessive expression of anger or frustration when playing with friends					
27. Participates in social games according to game rules, or those set by the group, e.g. during a ball game					
28. Solves problems that arise during a social game, e.g. when in disagreement					
29. When there is a conflict with a friend, thinks of multiple responses before responding, e.g. says it bothers him or calls for help					
30. Thinks about his own reactions when referring to friend's actions					

© All rights reserved
